# Versorgungsrealität der stationären vasoaktiven Therapie mit Prostazyklinderivaten bei Patienten mit akralen Durchblutungsstörungen bei systemischer Sklerose in Deutschland

**DOI:** 10.1007/s00393-019-00743-9

**Published:** 2020-02-10

**Authors:** A. Juche, E. Siegert, U. Mueller-Ladner, G. Riemekasten, C. Günther, I. Kötter, J. Henes, N. Blank, R. E. Voll, J. Ehrchen, M. Schmalzing, L. Susok, T. Schmeiser, C. Sunderkoetter, J. Distler, M. Worm, A. Kreuter, O. N. Horváth, M. P. Schön, P. Korsten, G. Zeidler, C. Pfeiffer, T. Krieg, N. Hunzelmann, P. Moinzadeh

**Affiliations:** 1 Klinik für Rheumatologie, Immanuel Krankenhaus Berlin-Buch, Berlin, Deutschland; 2 Klinik für Rheumatologie u. klinischer Immunologie, Charité Berlin, Berlin, Deutschland; 3 Rheumatologie und klinische Immunologie, Kerckhoff-Klinik, Bad Nauheim, Deutschland; 4 Klinik für Rheumatologie und Immunologie, Universitätsklinikum Schleswig-Holstein, Lübeck, Deutschland; 5 Klinik und Poliklinik für Dermatologie, Universitätsklinikum Carl Gustav Carus an der Technischen Universität Dresden, Dresden, Deutschland; 6 Klinik für Rheumatologie, klinische Immunologie u. Nephrologie, Asklepios Kliniken Hamburg, Hamburg, Deutschland; 7 Zentrum für interdisziplinäre Rheumatologie, Immunologie und Autoimmunerkrankungen INDIRA und Medizinische Klinik II, Universitätsklinik Tübingen, Tübingen, Deutschland; 8 Medizinische Klinik f. Hämatologie, Onkologie u. Rheumatologie, Universitätsklinikum Heidelberg, Heidelberg, Deutschland; 9 Klinik für Rheumatologie u. Klinische Immunologie, Medizinische Fakultät, Universität Freiburg, Freiburg, Deutschland; 10 Klinik für Hautkrankheiten, allg. Dermatologie u. Venerologie, Universitätsklinikum Münster, Münster, Deutschland; 11 Rheumatologie/Klinische Immunologie, Medizinische Klinik II, Universitätsklinikum Würzburg, Würzburg, Deutschland; 12 Klinik für Dermatologie, Allergologie u. Venerologie der Ruhr-Universität Bochum, Krankenhaus St. Josef-Hospital Bochum, Bochum, Deutschland; 13 Klinik für Rheumatologie, Immunologie u. Osteologie, St. Josef Wuppertal, Wuppertal, Deutschland; 14 Universitätsklinik u. Poliklinik für Dermatologie u. Venerologie, Universitätsklinikum Halle (Saale), Halle (Saale), Deutschland; 15 Medizinische Klinik für Rheumatologie u. Immunologie, Universitätsklinikum Erlangen, Erlangen, Deutschland; 16 Klinik für Dermatologie, Venerologie u. Allergologie, Charité Berlin, Berlin, Deutschland; 17 Klinik für Dermatologie, Venerologie und Allergologie, HELIOS St. Elisabeth Klinik Oberhausen, Universität Witten/Herdecke, Oberhausen, Deutschland; 18 Klinik für Dermatologie u. Allergologie, Ludwig-Maximilians Universität München, München, Deutschland; 19 Klinik für Dermatologie, Venerologie u. Allergologie, Universitätsmedizin Göttingen, Göttingen, Deutschland; 20 Niedersächsisches Institut für Berufsdermatologie, Universitätsmedizin Göttingen, Göttingen, Deutschland; 21 Klinik für Nephrologie u. Rheumatologie, Universitätsmedizin Göttingen, Göttingen, Deutschland; 22 Klinik für internistische Rheumatologie, Orthopädie u. Rheumachirurgie, Johanniter-Krankenhaus im Fläming, Treuenbrietzen, Deutschland; 23 Klinik für Dermatologie u. Allergologie, Universitätsklinikum Ulm, Ulm, Deutschland; 24 Klinik und Poliklinik für Dermatologie und Venerologie, Universität zu Köln, Köln, Deutschland

**Keywords:** SSc, Sklerodermie, Iloprost, Raynaud, Digitale Ulzera, Systemic sclerosis, Scleroderma, Iloprost, Raynaud’s phenomenon, Digital ulceration

## Abstract

**Hintergrund:**

Das Raynaud-Phänomen und die damit häufig einhergehenden digitalen Ulzerationen stellen für Patienten mit systemischer Sklerose (Sklerodermie [SSc]) ein frühes und sehr belastendes Symptom mit bedeutenden Einschränkungen der Arbeitsfähigkeit und Lebensqualität dar. Der Einsatz vasoaktiver Medikamente (insbesondere intravenöser Prostazyklinderivate) soll helfen, das Risiko hypoxischer Gewebeschäden bis hin zum Verlust der Finger zu reduzieren.

**Methoden:**

Um Aufschluss über die aktuelle Versorgung von Patienten mit Prostazyklinderivaten im klinischen Alltag in Deutschland zu erhalten, führten wir eine Umfrage unter den im Deutschen Netzwerk für systemische Sklerodermie (DNSS) zusammengeschlossenen Kliniken durch. Zusätzlich erfolgte eine separate Patientenbefragung über die Sklerodermie Selbsthilfe e. V., die sich nur auf die Symptome „Raynaud-Phänomen“ und „Digitale Ulzera“ und den Einsatz intravenöser Prostazyklinderivate bezog.

**Ergebnisse:**

Von den befragten 433 Patienten gaben 56 % an, dass sie bereits aufgrund ihrer Erkrankung und Symptome mit Prostazyklinderivaten behandelt wurden. Insgesamt 61 % erhielten die Therapie aufgrund starker Raynaud-Symptomatik und 39 % aufgrund digitaler Ulzerationen. Die meisten Befragten erfuhren durch die Therapie nicht nur eine Verbesserung des Raynaud-Phänomens und der digitalen Ulzera, sondern auch eine wesentliche Verbesserung von Einschränkungen im Alltag. Sie gaben zudem an, wesentlich weniger fremde Hilfe in Anspruch genommen sowie wesentlich weniger Fehlzeiten bei der Arbeit gehabt zu haben.

**Schlussfolgerung:**

Die Patienten empfanden durchweg einen positiven Effekt der Therapie mit Prostazyklinderivaten auf das Raynaud-Phänomen, ihre digitalen Ulzerationen, Schmerzen und Alltagseinschränkung und fühlten sich durch die stationäre Therapie gut und sicher betreut. Diese positiven Effekte in der Patientenwahrnehmung sind eine eindrückliche Stütze und bestätigen nachdrücklich die auf europäischer und internationaler Ebene erarbeiteten Therapieempfehlungen.

**Zusatzmaterial online:**

Die Online-Version dieses Beitrags (10.1007/s00393-019-00743-9) enthält die 2 Fragebögen, die für die Befragung verwendet wurden. Beitrag und Zusatzmaterial stehen Ihnen auf www.springermedizin.de zur Verfügung. Bitte geben Sie dort den Beitragstitel in die Suche ein, das Zusatzmaterial finden Sie beim Beitrag unter „Ergänzende Inhalte“.

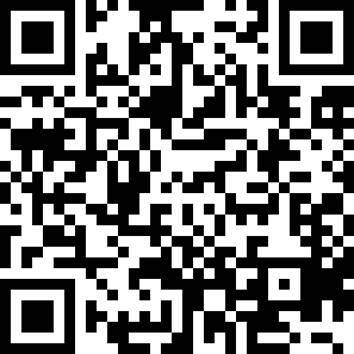

## Abstract (en)

**Background:**

Raynaud’s phenomenon and the frequently ensuing digital ulcerations represent an early and very distressing symptom in patients with systemic sclerosis (scleroderma, SSc) causing significant limitations in the ability to work and quality of life. The use of vasoactive drugs (especially intravenous prostacyclin derivatives) is recommended to reduce the risk of hypoxic tissue damage up to the loss of fingers.

**Methods:**

In order to obtain information about the current state of treatment of patients with prostacyclin derivatives in routine clinical life in Germany, a survey was conducted among the centers affiliated to the German Network for Systemic Scleroderma (DNSS). In addition, a separate patient survey was conducted by the schleroderma self-help group (*Sklerodermie Selbsthilfe e.* *V*.), which only covered the symptoms Raynaud’s syndrome, digital ulcers and the use of intravenous prostacyclin derivatives.

**Results:**

Of the 433 patients surveyed 56% stated that they had already been treated with prostacyclin derivatives (iloprost/alprostadil) because of their illness and symptoms. A total of 61% received the treatment for severe Raynaud’s phenomenon and 39% for digital ulcerations. Most respondents not only experienced an improvement in Raynaudʼs phenomenon and digital ulcers but also a significant improvement of limitations in everyday life. They also needed significantly less outside help and absenteeism from work was much lower.

**Conclusion:**

Patients consistently reported a positive effect of treatment with prostacyclin derivatives on Raynaudʼs phenomenon, acral ulcerations, pain and daily restrictions and felt well and safely cared for during inpatient treatment. These positive effects in the patientsʼ perceptions provide crucial information supporting and confirming the current European and international treatment recommendations.

## Einleitung und Hintergrund

Die systemische Sklerose (SSc) ist eine Multisystemerkrankung, die sich nicht nur an der Haut, sondern auch kardiopulmonal, renal, gastrointestinal und am Bewegungsapparat manifestieren kann [4]. Das sekundäre Raynaud-Syndrom (Raynaud-Phänomen [RP]), das bei mehr als 93 % der Patienten als [15] initiales Symptom vorhanden ist, kann – je nach Subtyp – den weiteren Organbeteiligungen Jahre bis Jahrzehnte vorausgehen und die Patienten in ihrer Lebensqualität massiv beeinträchtigen. Aufgrund rezidivierender Attacken können hypoxische Gewebeschäden auftreten, die sich in Ulzerationen, Fissuren, „pitting scars“ oder digitalen Nekrosen äußern. Zu den gefürchteten Komplikationen digitaler Ulzerationen zählen Superinfektionen, trockene oder feuchte Gangrän, Amputationen betroffener Fingerspitzenkuppen und damit einhergehende Schmerzen und Einschränkungen der Handfunktion im täglichen Leben [7, 12]. Eine relativ hohe Anzahl an SSc-Patienten (43–48 %) entwickelt im Verlauf ihrer Erkrankung digitale Ulzerationen [1], für deren Auftreten ein früher Beginn des RP ein Risikofaktor darstellt [6, 20] und die mit gesteigerten kardiovaskulären Komplikationen und einem verminderten Überleben assoziiert sind [13].

Zur Verminderung von Raynaud-Attacken und zur Vorbeugung digitaler Ulzerationen empfiehlt man den Patienten einen konsequenten Kälteschutz und physikalische Maßnahmen (isolierende, heizbare Handschuhe, Schuhsohlen, Taschenwärmer, Paraffinbäder, beheizbare Hirsekissen) sowie das Meiden von Verletzungen und Nikotinkonsum [18]. Da jedoch diese unterstützenden Maßnahmen alleine meist nicht ausreichen, ist es wichtig, frühzeitig durch systemische gefäßerweiternde Therapiestrategien einzugreifen. Neben Kalziumkanalblockern und Angiotensin-II-Rezeptor-1-Antagonisten stehen dafür heute intravenös (i.v.) applizierbare Prostazyklinderivate (z. B. Iloprost, Alprostadil) zur Verfügung, die die therapeutischen Möglichkeiten in der Behandlung und Prävention von schweren Ischämien, Gangrän und Amputationen deutlich verbessert haben [8, 22].

Der Endothelin-Rezeptorantagonist Bosentan ist für die Prävention von akralen Ulzerationen zugelassen, hat jedoch keinen wesentlichen Effekt auf die Abheilung bestehender Ulzerationen [9–11].

Eine kurative Therapie für Patienten mit SSc existiert bis heute nicht. Daher ist es entscheidend, frühzeitig die entstehenden Symptome und Organmanifestationen gezielt zu behandeln, um den Gewebeschaden so gering wie möglich zu halten. International gibt es klare Empfehlungen zur Behandlung des Raynaud-Syndroms und digitaler Ulzerationen. Gut etabliert sind die im Jahr 2017 aktualisierten European League against Rheumatism(EULAR)-Therapierichtlinien, die bei digitalen Ulzerationen die Gabe von intravenös appliziertem Iloprost empfehlen. Da meist eine orale Therapie mit Kalziumkanalblockern oder Angiotensin-II-Rezeptor-1-Antagonisten nicht ausreicht, sollten heute auch beim schweren sekundären Raynaud-Syndrom Prostazyklinderivate wie Iloprost angewendet werden [8]. Auch Phosphodiesterase-5-Inhibitoren wie Sildenafil können zur Anwendung kommen, sind jedoch für die Behandlung des Raynaud-Phänomens und digitaler Ulzerationen nicht zugelassen [5, 10].

Eine ganz aktuelle Studie (PROSIT) macht noch einmal ganz deutlich, dass Iloprost Mittel der ersten Wahl bei ausgeprägtem Raynaud-Syndrom und digitalen Ulzera ist [16]. Es existieren weltweit weiterhin unterschiedliche Schemata bezüglich der Häufigkeit der Iloprost-Gabe. Allerdings ist in vielen Studien klargestellt worden und es besteht Konsens (nach Delphi-Konsensus-Verfahren), dass Iloprost zur Therapie digitaler Ulzerationen und zur Prävention neuer digitaler Ulzerationen bei Patienten mit entsprechender Anamnese oder erhöhtem Risiko zur Anwendung kommen sollte [7].

## Wirkmechanismus und Nebenwirkungsspektrum von Prostazyklinderivaten

Insgesamt kommen in Deutschland 2 unterschiedliche Prostazyklinderivate zur Anwendung.

Bei *Iloprost* handelt es sich um ein synthetisches Analogon des Prostazyklin (Prostaglandin I_2_). Iloprost verbessert die arterielle Durchblutung durch eine Vasodilatation, hemmt die Thrombozytenaggregation und -adhäsion, wirkt zugleich entzündungshemmend und moduliert die Expression von Adhäsionsmolekülen auf Endothelzellen. Derzeit ist Iloprost als Infusion zur Therapie der Thrombangiitis obliterans und fortgeschrittener, schwerer Durchblutungsstörungen sowie als Inhalat zur Therapie der idiopathischen arteriell-pulmonalen Hypertonie zugelassen [2]. Aufgrund des Wirkungsmechanismus wird es zudem zur Therapie des Raynaud-Syndroms und damit einhergehenden digitalen Ulzerationen eingesetzt.

*Alprostadil* ist ein Prostaglandin-E-Derivat (Prostaglandin E1). Eine Indikation besteht für die chronische arterielle Verschlusskrankheit im Stadium III und IV, wenn eine Lumenerweiterung nicht möglich oder erfolglos ist. In der Behandlung der arteriellen Verschlusskrankheit spielen möglicherweise auch fibrinolytische Wirkungen und eine Hemmung der Leukozytenaktivierung eine Rolle. Zusätzlich wirken Prostaglandine vasodilatierend, hemmen die Thrombozytenaggregation und stimulieren die intestinale glatte Muskulatur.

*Unerwünschte Wirkungen* der Prostazyklinderivate ähneln sich. Häufig sind Kopfschmerzen, Flush, Übelkeit und Erbrechen. Es kann zu schwerwiegenden Komplikationen kommen, zum Teil durch ausgeprägte Hypotonie ausgelöst, unter anderem Herzinfarkt, Lungenödem oder Herzversagen. Daher ist ein engmaschiges Monitoring während und nach der Applikation erforderlich. Eine Aufstellung der häufigsten unerwünschten Wirkungen und Kontraindikationen findet sich in Tab. 1 [2].

**Table 1:** 

Häufigkeit	Nebenwirkungen
Sehr häufig (>10 %)	Brechreiz, Erbrechen, Flush (58 %), Hyperhidrosis, Kopfschmerzen (69 %), Magen-Darm-Störung (30 %), Übelkeit
Häufig (1–10 %)	Appetitlosigkeit, Arthralgie, Benommenheit, Blutdruckabfall, Bradykardie, Diarrhö, Durst, Erregungszustand, Erschöpfung, Erythem bzw. Phlebitis an der Injektionsstelle, Fieber, Hitzegefühl, Hypotonie, Kieferschmerz, Lethargie, Magenschmerz, Müdigkeit, Muskelschmerz, Muskelschwäche, Parästhesien, lokale und allgemeine Schmerzen, Schüttelfrost, Schwächezustand, Schwindel, Trismus, Unruhezustand, Unwohlsein
Kontraindikation	–

Überempfindlichkeitsreaktionen, schwere koronare Herzkrankheit/instabile Angina pectoris, Herzrhythmusstörung, Herzinsuffizienz (NYHA II–IV), Zustand nach Myokardinfarkt innerhalb der letzten 6 Monate, bei erhöhtem Blutungsrisiko (im Rahmen von schweren Verletzungen, Ulcus ventriculi, Gehirnblutung), Schwangerschaft, Stillzeit, bei Verdacht auf Lungenstauung

## Studiendesign und Methodik der Befragung

**Figure 1:**
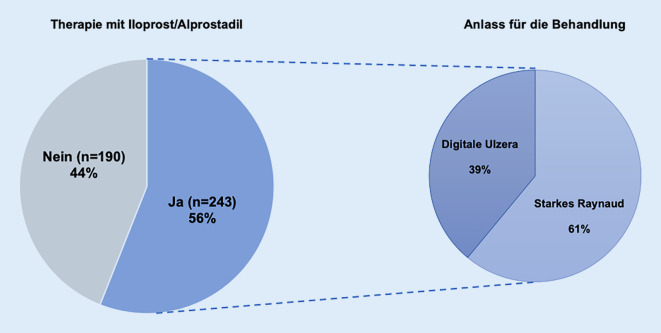

**Figure 2:**
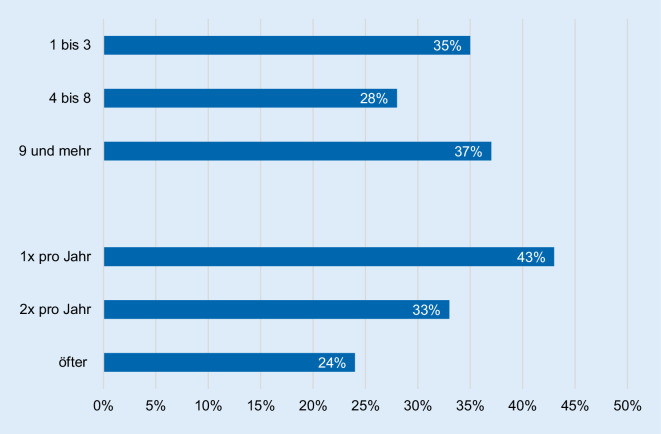

Wir führten 2 voneinander getrennte Befragungen zur Behandlung mit Prostazyklinderivaten durch. Zum einen erfolgte eine Befragung der Zentren/Ärzte des Deutschen Netzwerks für Systemische Sklerodermie e. V. (DNSS) mittels Fragebogen (Papierform) (s. Electronic Supplement Material Abb. 1), zum anderen eine Befragung der Patienten über die Sklerodermie Selbsthilfe e. V. (s. Electronic Supplement Material Abb. 2), die sowohl online als auch in Papierform in der Zeit von Oktober 2018 bis März 2019 durchgeführt wurde. Die Patientenbefragung bezog sich nur auf die Symptome „Raynaud-Syndrom“ und „digitale Ulzera“ und den Einsatz von Prostazyklinderivaten. Insgesamt 21 klinische Zentren und 433 Patienten haben an der Befragung teilgenommen. Es handelt sich um eine quantitative Befragung; zusätzlich konnten Patienten und Kliniker auch individuelle Anmerkungen und Erklärungen anfügen, welche bei der Auswertung der Ergebnisse ebenfalls berücksichtigt wurden.

Bei den Befragungen der DNSS-Zentren und der Patienten wurden 2 separate Fragebögen eingesetzt. Die befragten Ärzte gehören Zentren an, die dem DNSS angeschlossen sind. Die Befragung der Patienten über die Sklerodermie Selbsthilfe e. V. erfolgte deutschlandweit und erreichte somit auch Patienten, die nicht notwendigerweise an DNSS-Zentren betreut werden.

Die gestellten Fragen an die behandelnden Ärzte betrafen die Versorgungssituation betreuter Patienten mit i.v.-Prostazyklinderivaten. Hier wurde erfasst, ob Prostazyklinderivate eingesetzt werden, welches Präparat verwendet wird, ob die Gabe (teil)stationär oder ambulant erfolgt, bei welcher Indikation es verabreicht wird und ob bereits Probleme mit der Kostenübernahme entstanden sind. Diese Daten wurden vom DNSS gesammelt und ausgewertet (SPSS, 23.0.0.3 64-Bit, IBM Corp., Armonk, NY, USA, und Microsoft Excel, Microsoft Corp., Redmond, WA, USA).

Die Befragung der Patienten bezog sich auf die Qualität der Betreuung, Nebenwirkungen der Therapie, Verringerung von Schmerzen, Verringerung von Raynaud-Beschwerden und Einschränkungen im Alltag. Die Datensammlung/‑analyse der Patienten erfolgte mithilfe von Admedicum® Business for Patients GmbH & Co KG und Microsoft Excel (Microsoft Corp., Redmond, WA, USA). Manche Fragen konnten anhand vorgegebener Antwortmöglichkeiten beantwortet werden, andere anhand angepasster Skalen, die von 1 bis 10 zu beantworten waren.

Das Ziel dieser Befragung ist, die aktuelle Versorgungsrealität mit i.v.-Prostazyklinderivaten von Patienten mit systemischer Sklerose und akralen Durchblutungsstörungen darzustellen.

## Verordnungssituation bei Anwendung von Prostazyklinderivaten in Deutschland – Sicht der Zentren/Ärzte

Da diverse internationale und europäische Empfehlungen/Richtlinien zur Therapie vaskulärer Symptome bei Patienten mit SSc existieren, sollte auch die Verordnungssituation in Deutschland im Rahmen des Deutschen Netzwerks für Systemische Sklerodermie (DNSS) untersucht werden. Die Zentren des DNSS zeichnen sich durch besondere Expertise und Kompetenz in der Betreuung und Therapie von Patienten mit Sklerodermie aus.

Um Aufschluss über die aktuelle Versorgung von Patienten mit Prostazyklinderivaten im klinischen Alltag (stationär vs. ambulant) in Deutschland zu erhalten, führten wir eine Umfrage unter den im DNSS zusammengeschlossenen Kliniken durch. Diese betreuen ca. 80 % aller Patienten in Deutschland.

Insgesamt 81 % (21/26) der angefragten DNSS-Zentren antworteten. Dabei zeigte sich, dass eine Infusionsbehandlung mit vasoaktiven Substanzen (Prostazyklinderivate) derzeit nur im stationären Rahmen erfolgt (stationär deutlich häufiger (86 %) als teilstationär (5 %)), und dies meist mit Iloprost (76 %) (Tab. 2). Bezüglich der Indikation gaben 91 % (19/21) der befragten Zentren an, es bei schwerem Raynaud-Phänomen zu verabreichen, 81 % (17/21) bei sekundärem Raynaud-Phänomen und auch 33 % (7/21) bei primärem Raynaud-Phänomen. Alle Zentren verabreichen Prostazyklinderivate bei digitalen Ulzerationen ohne Superinfektion (21/21), 86 % (18/21) bei digitalen Ulzerationen mit Superinfektion und 76 % (16/21) bei beginnender Gangrän (Mehrfachantworten bei dieser Frage möglich). Ein Drittel der Zentren gaben an, bei (teil) stationärer Gabe der Prostazyklinderivate Probleme mit Kostenträgern zu haben.

**Table 2:** 

Eingesetzte Therapien und Applikationsform	21 Zentren; % (*n*)
Iloprost	76; (16/21)
Alprostadil	10; (2/21)
Beide Therapie (Iloprost und Alprostadil)	14; (3/21)
Nur stationäre Gabe	86; (18/21)
Nur teilstationäre Gabe	5; (1/21)
Ambulante Gabe	0; (0/21)
Stationäre und teilstationäre Gabe	10; (2/21)

## Verordnungssituation bei Anwendung von Prostazyklinderivaten in Deutschland – Sicht der betroffenen Patienten

Insgesamt 44 % (190/433) der befragten Patienten wurden bisher noch nie mit Präparaten aus einer der beiden Substanzklassen behandelt und daher bei der Datenanalyse nicht mehr weiter einbezogen.

Hingegen gaben 56 % (243/433) an, dass sie bereits aufgrund ihrer Erkrankung und Symptome mit Prostazyklinderivaten behandelt werden mussten. Von diesen bekamen 61 % die Therapie aufgrund starker Raynaud-Symptomatik und 39 % aufgrund digitaler Ulzerationen. Insgesamt 10 % der Patienten gaben an, sowohl aufgrund starker Raynaud-Beschwerden als auch aufgrund digitaler Ulzerationen behandelt worden zu sein (Abb. 1).

Die meisten Patienten erhielten Iloprost (76 %, 183/233), 17 % (40/233) Alprostadil, 3 % (6/233) beide Präparate, und 2 % (4/233) gaben an, ein anderes Präparat erhalten zu haben.

Die Mehrheit der Befragten hatte bereits mehrere Infusionszyklen erhalten und Therapieerfahrungen sammeln können. Zusätzlich wurde von den Patienten beschrieben, dass die Infusionen in ihrer Häufigkeit sowohl an den individuellen Gesundheitszustand als auch an die Jahreszeit angepasst verabreicht wurden (Abb. 2).

Die Infusionen wurden bei 81 % (189/233) der Patienten unter stationären Bedingungen appliziert, und bei weniger als 19 % erfolgte die Gabe ambulant (14 % ambulant in der Klinik und 5 % ambulant außerhalb des Krankenhauses).

## Betreuung und Nebenwirkungen der Therapie – Sicht der Patienten

In Bezug auf die *Betreuung* während der Verabreichung der Therapie fühlten die Betroffenen sich überwiegend sehr gut bis gut betreut (Mittelwert = 7,62 ± 2,3), unabhängig von der Indikation (schweres Raynaud-Phänomen und digitale Ulzerationen).

Bei der Befragung nach *Nebenwirkungen* gab die Mehrheit der befragten Patienten keine oder nur geringe Nebenwirkungen durch die Therapie an (Mittelwert ± SD = 4,02 ± 2,5). Patienten mit Raynaud-Syndrom empfanden etwas weniger Nebenwirkungen als Patienten, die aufgrund digitaler Ulzerationen behandelt wurden. Laut der Befragung sei auf Nebenwirkungen beim Großteil der Befragten sehr gut oder gut reagiert worden (Mittelwert ± SD = 7,71 ± 2,6). Bei der Unterscheidung nach Diagnose gab es keine nennenswerten Unterschiede.

## Effekt der Therapie auf das Raynaud-Syndrom, die digitalen Ulzera und damit einhergehende Schmerzen – Sicht der Patienten

**Figure 3:**
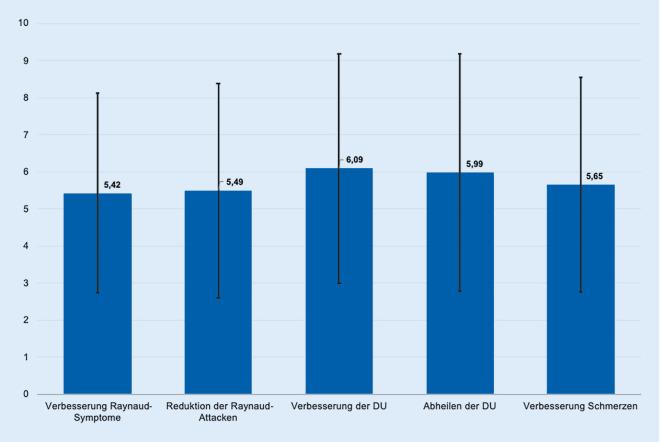

Den *Therapieeffekt* empfanden die behandelten Patienten sowohl beim Raynaud-Syndrom als auch bei digitalen Ulzerationen sehr ähnlich (Abb. 3). Der Großteil der befragten Patienten gab an, unter der Therapie eine mäßige bis gute *Verbesserung der Raynaud-Symptome* erfahren zu haben (Mittelwert ± SD = 5,42 ± 2,7). Patienten, die keine Raynaud-Symptome hatten, haben die Antwortoption „1 – keine Besserung“ gewählt, da bei nicht vorhandener Symptomatik von keiner Besserung ausgegangen werden kann. Die *Häufigkeit der Raynaud-Attacken* konnte durch die eingesetzte Therapie mäßig bis stark reduziert werden (Mittelwert ± SD = 5,49 ± 2,9).

Die meisten Patienten erfuhren unter der Therapie eine mäßige bis sehr gute *Verbesserung der digitalen Ulzera* (Mittelwert ± SD = 6,09 ± 3,1). Auch hier wählten die Patienten, die keine digitalen Ulzera hatten, die Antwortoption „1 – keine Besserung“, da bei nicht vorhandener Symptomatik von keiner Besserung ausgegangen werden kann.

Unter der Therapie erfuhren die meisten Patienten eine mäßig schnellere bis sehr gute Abheilung der digitalen Ulzera (Mittelwert ± SD = 5,99 ± 3,2).

In Bezug auf empfundene *Schmerzen *schilderten die meisten Patienten eine deutliche Linderung der Schmerzen (Mittelwert ± SD = 5,65 ± 2,9) (Abb. 3).

## Effekt der Therapie auf Einschränkungen im Alltag, Notwendigkeit fremder Hilfe sowie Fehlzeiten

**Figure 4:**
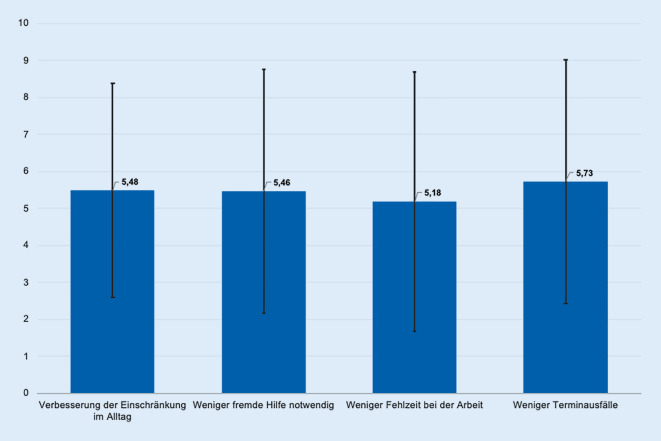

Die meisten Befragten erfuhren durch die Therapie eine Verbesserung von Einschränkungen im Alltag (Mittelwert ± SD = 5,48 ± 2,9) und gaben zudem an, wesentlich weniger fremde Hilfe in Anspruch genommen (Mittelwert ± SD = 5,46 ± 3,3) sowie wesentlich weniger Fehlzeiten bei der Arbeit gehabt zu haben (Mittelwert ± SD = 5,18 ± 3,5) (Abb. 4).

## Bisherige Studienlage zu Prostazyklintherapien bei systemischer Sklerose

Ein 1998 vorgelegtes systematisches Cochrane-Review zur Frage der Wirksamkeit vasoaktiver Therapien mit Prostazyklinen bei systemischer Sklerose [17] wurde 2017 durch eine systematische Literaturrecherche im Rahmen der Publikation einer EULAR-Leitlinie zur Behandlung der systemischen Sklerose [8] mit einem Recherchezeitraum von 1966 bis September 2014 ergänzt. Schließlich umfasst die Literaturrecherche eines Konsensusartikels zur Therapie mit Iloprost von Ingegnoli et al. [7] einen Suchzeitraum von Januar 1950 bis Januar 2017. Wir führten eine erneute systematische Literaturrecherche in PubMed sowie dem Cochrane-Studienregister mit nachfolgender Suchangabe durch: („(prostanoids OR iloprost) AND (systemic sclerosis OR scleroderma) AND (digital OR raynaud) AND (trial OR study)“). Der Zeitraum umfasste Januar 2017 bis Mai 2019. Dabei sollten nur Metaanalysen von randomisiert kontrollierten Studien (RCTs), systematische Übersichten, RCTs, prospektive Fall-Kontroll- oder prospektive Kohortenstudien mit klinisch relevanten Endpunkten wie Abheilung digitaler Ulzera, Besserung von Häufigkeit oder Schwere der Raynaud-Attacken ausgewählt werden. Wir konnten allerdings keine zusätzlichen neuen Studien auffinden.

Das systematische Cochrane-Review zeigte, dass Iloprost die Häufigkeit und Schwere von Raynaud-Attacken signifikant verringern kann. Hinsichtlich Prävention und schnellerer Abheilung digitaler Ulzera ergaben kleinere RCTs signifikante Ergebnisse, dagegen eine Metaanalyse [21] nur einen Trend auf bessere Ergebnisse unter Iloprost.

Zu Alprostadil fanden wir nur 2 RCTs. Der größere mit 55 Patienten [14] zeigt keine Besserung von Raynaud-Beschwerden und Abheilung von Ulzera nach 14 bzw. 28 Tagen verglichen mit Placebo. Der kleinere mit lediglich 12 Patienten [3] berichtet über signifikante Besserung von Raynaud-Beschwerden.

In validen Vergleichsstudien kaum untersucht sind die unterschiedlichen Infusionsschemata der Prostazyklinderivate weltweit. Hierzu gibt es noch keinen allgemein akzeptierten Standard.

## Diskussion und Schlussfolgerung

Bei der chronischen Multisystemerkrankung systemische Sklerose (Sklerodermie) sind nicht nur die Beteiligung innerer Organe mit damit verbundenen Komplikationen ein großes Problem, ebenfalls belastend sind massive Schmerzen aufgrund der Raynaud-Attacken, insbesondere der dadurch bedingten digitalen Ulzerationen. Diese schränken die Alltagsaktivität vieler Patienten deutlich ein, es kommt zu massiven Schlafstörungen und bei Komplikationen zu septischen Verläufen und Fingeramputationen. Bei dem praktisch immer vorliegenden Raynaud-Syndrom gehören vasoaktive Therapien, insbesondere als Infusion mit Prostazyklinderivaten zur Standardbehandlung. Hierdurch konnten deutlich positive Verbesserungen und Erfolge erzielt werden, die die Lebensqualität vieler Patienten massiv verbessert haben. Der Nutzen dieser Behandlung bezüglich Besserung von Raynaud-Beschwerden oder schnellerem Abheilen digitaler Ulzera zeigt sich in vielen Studien und Metaanalysen, auch wenn die Dosierungen und die Therapieschemata in den verschiedenen Zentren weltweit noch unterschiedlich angewendet werden. Häufig angewandte Dosierungen von Iloprost betragen 0,5–2 ng/kg/min über 6–8 h. Hinsichtlich der Häufigkeit von Infusionen gibt es Hinweise aus nicht randomisierten Vergleichen, dass einmalige Infusionen in häufiger Folge, etwa monatlich, effektiver erscheinen als Infusionsserien über mehrere Tage hintereinander in größeren, etwa halbjährlichen oder jährlichen Abständen (zuletzt [19]). Valide Studien, die diese vorläufigen Ergebnisse bestätigen, stehen allerdings noch aus.

Trotz solider wissenschaftlicher Evidenz zum Nutzen der Infusionstherapie bestehen im Versorgungsalltag Verordnungsprobleme, wie unsere Umfrage eindrücklich zeigt. Eine Zulassung der Prostazyklinderivate liegt für andere vaskuläre Erkrankungen, nicht aber für die Behandlung des Raynaud-Syndroms bei Patienten mit systemischer Sklerose vor. Hierdurch ist die Finanzierung durch die Kostenträger zum Teil nur als Einzelfallentscheidung möglich. Da es sich um eine große Patientengruppe mit deutlicher Lebensqualitätseinschränkung und nachweisbarem Nutzen der Therapie und ohne zugelassene Vergleichstherapie handelt, ist dies für Arzt und Patienten eine schwierige Situation. Die Infusionstherapie dauert ca. 8 h oder mehr pro Tag. Neben seltenen ernsten unerwünschten Wirkungen treten oft Nebenwirkungen auf, die zwar gut beherrschbar sind, aber eine kontinuierliche Überwachung der Patienten notwendig machen. Erschwerend tritt hinzu, dass viele Patienten weitere vasoaktive Substanzen beispielsweise zur Therapie einer arteriellen Hypertonie oder einer pulmonalarteriellen Hypertonie (PAH) erhalten. So ist aus unserer Sicht eine stationäre Infusionsbehandlung für praktisch alle Patienten erforderlich. Die Erfahrungen in der praktischen Behandlung der systemischen Sklerose zeigen jedoch, dass in einem großen Teil diese unverzichtbare Therapie durch Krankenkassen mit dem formalen Hinweis auf die Möglichkeit einer ambulanten Gabe verhindert wird. Gerade aufgrund der bei diesen Patienten durch eine Myokardbeteiligung vermehrten Gefahr plötzlich auftretender Herzrhythmusstörungen ist eine Therapie ohne Überwachung auch nach den Infusionen nicht zu empfehlen.

Überzeugend ist in diesem Zusammenhang auch die in unserer Untersuchung erhobene große Zufriedenheit der Patienten mit dem Nutzen der Infusionstherapie mit einer langsamen Dosissteigerung. Die Patienten empfinden durchweg einen positiven Effekt auf Raynaud-Symptome, akrale Ulzerationen, Schmerzen und Alltagseinschränkung und fühlen sich durch das stationäre Therapiesetting gut und sicher betreut. Die kontinuierliche Überwachung gestattet eine schnelle Reaktion und Anpassung der verträglichen Dosierung, die den Patienten sehr entgegenkommt und zu einer Reduktion der oft deutlichen Nebenwirkungen führt und zusätzlich den Patienten die Angst vor Nebenwirkungen nimmt.

Die Analyse dokumentiert, dass die Patienten mit dieser Therapie deutlich weniger auf fremde Hilfe angewiesen sind, weniger Fehlzeiten bei der Arbeit und weniger Ausfälle bei vereinbarten Terminen aufweisen.

Limitationen der Patientenbefragung sind, dass die Studie retrospektiv Daten erfasst und per Fragebogen allgemeine Beurteilungen der Patienten über längere Zeiträume abfragt. Es wurden keine validierten bzw. standardisierten Outcome-Parameter erhoben. Somit ist diese Datenerhebung mit einer kontrollierten, prospektiven Studie mit standardisierten Outcome-Parametern nicht zu vergleichen.

Deshalb war auch ein direkter Vergleich zwischen Iloprost und Alprostadil nicht möglich. International wird die Gabe von Iloprost empfohlen und bevorzugt.

Die positiven Effekte in der Patientenwahrnehmung unterstützen nachdrücklich die europäischen und internationalen Therapieempfehlungen, die eine Therapie mit Prostazyklinderivaten empfehlen. Diese lassen sich für fast alle Patienten nur (teil)stationär realisieren. Die Kostenträger sind unserer Ansicht nach aufgerufen, betroffenen Patienten diese wertvolle Behandlung zugänglich zu halten.

## Caption Electronic Supplementary Material




