# Supplementary material for: Reality of inpatient vasoactive treatment with prostacyclin derivatives in patients with acral circulation disorders due to systemic sclerosis in Germany
Source: Z Rheumatol. 2020 Feb 10;79(10):1057–66. [Article in German] doi: 10.1007/s00393-019-00743-9 (PMC7708340; doi:10.1007/s00393-019-00743-9)
Supplement: Supplementary file 1 [file 393_2019_743_MOESM1_ESM.docx]

**Supplement Abbildung 1:** Fragebogen an die Zentren/Ärzte
